# The important role of whole-process computed tomography guidance for percutaneous gastrostomy in esophageal cancer patients who are unsuitable for or have had unsuccessful attempts with endoscopic and fluoroscopic gastrostomy

**DOI:** 10.1186/s12876-023-03040-7

**Published:** 2024-01-03

**Authors:** Xiang Geng, Qing Zhao, Hang Yuan, Hai-Liang Li, Chen-Yang Guo, Ting Yang, Wei-Jun Fan, Jung-Hoon Park, Xiao-Hui Zhao, Wen-Bo Zhu, Hong-Tao Hu

**Affiliations:** 1 Department of Minimally & Invasive Intervention, The Affiliated Cancer Hospital of Zhengzhou University, Henan Cancer Hospital, NO.127, Dongming Road, Zhengzhou, 450008 Henan Province China; 2 Department of Radiology, The Second People’s Hospital of Jiaozuo, NO.17, Minzhu South Road, Jiaozuo, 454150 Henan Province China; 3 Department of Minimally & Invasive Intervention, Sun Yat-sen University Cancer Center, NO.651, Dongfeng east Road, Guangzhou, 510000 Guangdong Province China; 4 Biomedical Engineering Rearch Center, Asan Institute for Life Sciences, Asan Medical Center, 88 Olympic-ro 43- gil, Seoul, 05505 Korea

**Keywords:** Esophageal cancer, Dysphagia, Feeding tube placement, Interventional radiological procedure, Refractoty, Percutaneous gastrostomy, Whole process CT guided

## Abstract

**Purpose:**

To explore the value of clinical application with the whole process computed tomography (CT) guided percutaneous gastrostomy in esophageal tumor patients.

**Materials and methods:**

A consecutive series of 32 esophageal tumor patients in whom endoscopic gastrostomy or fluoroscopy guided gastrostomy were considered too dangerous or impossible due to the esophagus complete obstruction, complicate esophageal mediastinal fistula, esophageal trachea fistula or severe heart disease. All of the 32 patients were included in this study from 2 medical center and underwent the gastrostomy under whole process CT guided.

**Results:**

All of the gastrostomy procedure was finished successfully under whole process CT guided and the technical success rate was 100%. The average time for each operation was 27 min. No serious complications occurred and the minor complications occurred in 3 patients, including local infection, severe hyperplasia of granulation tissue and tube dislodgment. There were no procedure related deaths.

**Conclusion:**

The technical success rate of whole process CT guided percutaneous gastrostomy is high and the complication is low. This technique can be used feasible and effectively in some special patients.

## Introduction

In patients with advanced esophageal tumor, most patients experienced nutritional failure mainly due to the dysphagia. Malnutrition is mainly due to reduced intake, nutrition management was used to supply energy, liquid, vitamin and mineral needs and, where possible, to prevent aspiration. Percutaneous endoscopic gastrostomy (PEG) and percutaneous fluoroscopic gastrostomy (PFG) are known as standard methods for making sure long-term enteral food intake in patients with symptoms of dysphagia [1–3]. But we found some patients in clinic practice, the endoscopic gastrostomy or fluoroscopy guided gastrostomy were considered too dangerous or impossible due to the esophagus complete obstruction, complicate esophageal mediastinal fistula, esophageal trachea fistula or severe heart disease [4].

Some authors had been reported the method of computed tomography (CT) guidance or CT fluoroscopy-guided percutaneous gastrostomy [3, 5, 6]. In these reports, the patients underwent the insufflation by a nasal tube in the gastric lumen and then finished the gastrostomy by CT guided. But this method only can be performed for patients who can be inserted the nasal tube in the gastric lumen. If the patents cannot finish the insufflation, the CT guided percutaneous gastrostomy cannot be finished. Because of no radiation, ultrasound guidance is considered safer for patients. Church has reported gastrostomy tube placement by ultrasound-guided. However, before the percutaneous puncture, stomach was also needed to fill with saline through the orogastric tube [7]. Hu and Church reported using a 21-gauge needle puncture and finished the jejunostomy and gastrostomy [8, 9]. Inspired by these, thinking for these dangerous or impossible patients, we can percutaneous the stomach for insufflation using 21-gauge needle and do not need insufflate the stomach by insertion a nasal tube. In here, we reported our experience in 32 special patients who were performed gastrostomy under CT guided in whole procedure including the stomach lumen insufflation.

## Materials and methods

Institutional ethics committee approval our research. Written informed consent was obtained from all patients or their authorized representatives.

### Patients and study design

We retrospectively reviewed 32 esophageal tumor patients in whom were considered too dangerous or impossible by endoscopic or fluoroscopy guided due to the esophagus complete obstruction, complicate esophageal mediastinal fistula, esophageal trachea fistula or severe heart disease were introduced to interventional department of the two hospitals(The Affiliated Cancer Hospital of Zhengzhou University and The Second People's Hospital of Jiaozuo) from Dec. 2012 to Feb. 2019. Patient information is obtained from the electronic medical record system. All of the 32 patients include 22 male and 10 females, the average age was 67 years old (42–84 years old), who underwent a percutaneous gastrostomy under CT guidance for ensure the long-term enteral nutrition. Out of 32 patients, 22 underwent this treatment at the affiliated cancer hospital of zhengzhou university, and 10 received this treatment at the second people's hospital of Jiaozuo. Twenty-one patients underwent the failed endoscopic or fluoroscopy guided gastrostomy in other hospital because of the complete obstruction of esophagus and transfer to our hospital to do the CT guided gastrostomy. Eight patients cannot insert a catheter into the stomach in other hospital due to the complicate esophageal mediastinal fistula or esophageal trachea fistula. Three patients underwent the severe sinus arrhythmia or supraventricular tachycardia during the gastroscopy and they refused the insertion a catheter from mouth into the stomach.

In all of the 32 patients, 29 cases were diagnosed as esophageal cancer by histological pathology and three patients were diagnosed as esophageal tumor by upper gastrointestinal angiography without histological pathology due to the severe heart disease. The surgery or esophageal stent placement was not performed in all of the patients due to the patients’ opinion or the neoplasm staging.

### The gastrostomy procedure

Coagulation function is confirmed to be normal range. Antibiotics were not routinely used during the perioperative period. Drugs for sedation were not needed. All patients underwent the procedure under local anesthesiaTo inhibit stomach motility, an anticholinergic antispasmodic agent of anisodamine (Zhaohui, Shanghai, China) was administrated intramuscularly 30 min before the procedure.

The gastrostomy was performed by whole process CT guided (HiSpeed, General Electric Medical Systems, US) and the gastrostomy kit was used (15Fr, Create Medic, Japan). The percutaneous gastrostomy suit contains a loop fixture device for gastropexy, a trocar with peel-away sheath, a silicone balloon tube and 3 threads for suturing. There is a sliding plate element with two needles in gastropexy device: the yellow needle for inserting the gastropexy suture and the blue needle for inserting the retrieval loop and pulling out the suture. The solid steel 16Fr/17.5 cm trocar located in the inner of the 16Fr peel-away sheath and the sheath permit the tube insertion. The 15Fr gastrostomy tube with a small balloon (3 ml) for reducing the balloon shedding.

**Fig. 1:**
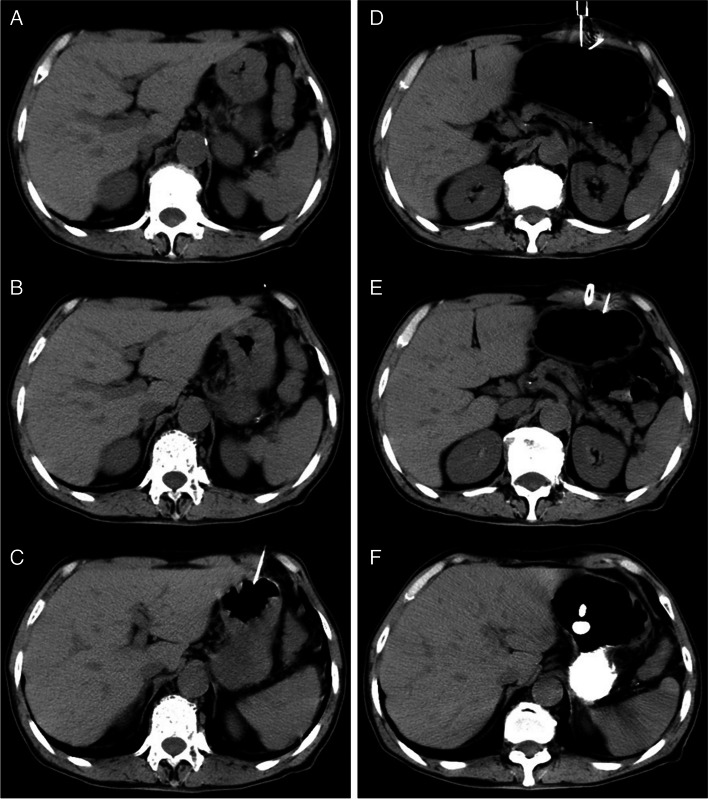
A 56-year old man who cannot underwent the gastrostomy under DSA guided due to the esophagus complete obstruction was referred to our department to undergo a CT guided percutaneous gastrostomy for long-term intragastric feeding. **A** A prepared abdominal CT scan was performed to localize the stomach and design the puncture pathway. **B** A metallic marker was placed on the skin surface to show the puncture pathway. **C** A 21-gauge Chiba needle was introduced into the gastric lumen and then the air inflation of gastric lumen was performed through the 21-gauge Chiba puncture needle. **D** After the stomach lumen insufflations finished, puncture procedure was performed with the gastropexy device. The complete gastropexy device was removed, maintaining traction on the wire loop retrieving the suture, which was released and tied in a standard surgical knot, opposing the anterior stomach to the anterior abdominal wall. **E** After successful gastropexy at both marked points a stab incision of about 5 mm was performed in between to insert the trocar with peel-away sheath carefully under CT guidance. The CT scan showed the trocar with peel-away sheath and the 21 G Chiba needle. **F** Once correct position was confirmed the trocar was removed and the balloon tube was inserted into the gastric lumen. The CT scan showed the balloon tube filled with 3 ml of contrast medium through an extra valve on the side using a syringe.

In all of the patients, images were acquired using 5-mm cuts to gastric lumen and then using 10-mm cuts after the gastric lumen inflation finished. The patients lay supine on a CT bed and underwent local anesthesia with 1% lidocaine. A prepared abdominal CT scan was performed to design the puncture pathway (Fig. 1 A) and then place a metal marker on the skin surface. Another CT scan to check the metal markers and measure the distance and angle between the stomach and the metal markers on the skin surface (Fig. 1 B). When the patient was in a breath hold, a 21-gauge Chiba needle (Chiba needle; Cook, Bloomington, USA) was used to puncture the stomach. after the puncture, a CT scan to check the position of the needle and adjust the needle if necessary (Fig. 1 C). A small amount of water-soluble contrast agent (Telebrix 300; Laboratoire Andre Guerbet) was injected through the needle to confirm the position of the needle in the stomach cavity. Normal air inflation of gastric lumen was performed through the 21-gauge Chiba puncture needle after confirming that the needle was in the right position. The gastric cavity expansion was observed by CT scan of the upper abdomen. After the stomach lumen insufflations finished, the following procedure and technique was performed as before reported in detail [5]. Briefly, two suitable sites for the puncture were determined by CT scan. After skin disinfection and local anesthesia, puncture was performed with the gastropexy device. The gastropexy device with two needles were vertically inserted into the gastric cavity at the first mark (Fig. 1 D). The contrast agent was injected and then a CT scan was performed to ensure that both needles reach the stomach cavity. Next, advanced the blue handle to open the retrieval loop, and inserted the gastropexy suture through the retrieval loop after removed the yellow trocar. Exited the blue handle and removed the complete gastropexy device with the gastropexy suture in the retrieval loop, then released the suture and tied it to the anterior abdominal wall. Repeated the above operations at the second puncture point. Made a 5 mm incision between the two marked points after successful gastropexy, and a trocar with peel-away sheath was carefully used to puncture under CT guidance (Fig. 1 E). Once the proper position was reached, removed the trocar and advanced the balloon tube far enough to make sure the balloon leaving the sheath into the stomach cavity. Then 3 ml of sterile water was filled into the low-profile balloon (Fig. 1 F). removed the peel-away sheath by bending the handles and keep the balloon tube location. Cleaned the puncture site, applied pressure on the retaining plate and the tube was moderately pulled for the well plate position.

Antibiotics are not routinely used postprocedure. On the first day postprocedure, contrast medium is injected through the balloon tube for fluoroscopic imaging. If the balloon tube is in a good position, patients are allowed to start nutrition through the balloon tube.The gastric fistula tube is replaced every 6 months, and patients are informed to return to the hospital within 24 h if the gastric fistula tube dislodges for replacement.

### Follow-up and study endpoints

We conduct patient follow-up through inpatient stays or telephone calls to assess whether patients can successfully receive nutrition through the gastric fistula tube, with follow-up continuing until the patient's death or February 2022.

### Statistical analysis

Data analysis was performed using SPSS (version 22; SPSS). Count data is represented in ranges.

## Results

The whole process CT guidance for percutaneous gastrostomy with loop gastropexy and combine peel-away sheath trocar technique were performed in all of the 32 patients. The gastrostomy treatment by this technique was successful in all of the 32 patients (100%). The first step of stomach lumen insufflations through the 21-gauge Chiba puncture needle was succeeded in 28 patients and failed in 4 cases in the first time. The failed reason in 4 cases is that the injected air was retained in the abdomen cavity, not in the gastric lumen judged by CT image. These patients underwent the Chiba needle puncture again and stomach lumen insufflations successfully by CT guided. During the period, patients did not complain the discomfort and the gastrostomy were performed smoothly in them. The procedure time was defined as the time between local anesthesia and CT scan to confirm that the balloon tube was in the stomach. The average procedure time was 27 min (16–64 min). During the procedure time, no serious adverse events occurred, such as aspiration, asphyxiation, etc. On the first day postprocedure, after fluoroscopic imaging, the ability of patients to receive nutrition through the balloon tube is considered clinical success. All patients postprocedure were able to successfully receive nutrition through the balloon tube, with a clinical success rate of 100%.

During the follow-up time, there were no serious adverse events such as peritonitis, mediastinitis, gastric colonic fistula, persistent local bleeding, systemic blood loss, or any local infection requiring surgical intervention were observed. The minor complication presents in 3 patients. One patient underwent the local infection and were cured by local sterilization. Another patient underwent severe hyperplasia of granulation tissue, he was cured by local debridement and sterilization. One patient encountered the tube dislodgment who was cured by inserting a new balloon tube in the same day when he came back the hospital. During the following up, 24 patients died. One patient died within 30 days after the procedure due to the tumor progress and no relationship with the gastrostomy. Twenty-three patients died due to the tumor progress from 6 to 27 months.

## Discussion

PFG tube insertion can be used as an alternative when PEG fails or PEG is not suitable [10, 11]. Although PFG provides long-term enteral nutrition safely and effectively for patients [1–3, 12, 13], the serious complications had been reported in the literature [2, 10]. The major and minor complication rates associated with PFG were 5.9% and 7.8%, respectively [2, 10] Liver abscess after fluoroscopy-guided gastrostomy had been reported as it passed through the liver parenchyma when the PFG was placed [14, 15]. It is difficult to adequately distinguish the relationship between the stomach and left liver lobe and the inter positioned colon by fluoroscopy guidance. The factors associated with complications during PFG procedure are usually due to hepatomegaly, enlarged or irregular left hepatic lobe, ascites or colon between the stomach, anterior abdominal wall, and other anatomical reasons [2, 16]. Using the whole process CT guided method, we can avoid these complications and our results confirmed this.On the other hand, due to various reasons, if the placement of the nasogastric tube failed, the routine PFG becomes challenging [17]. Heberlein has been reported that the gastrostomy tube insertion performed without nasogastric tube and the stomach punctured by a 21-gaugeneedle under fluoroscopic guidance [17]. But in these patients, the gastric bubble can be seen or use gas-producing powder to swell the stomach before puncture. If the stomach cavity is empty or the patient refuses to use the gas-producing powder, the CT guidance should be thought as a better choice. According our experience, puncture a stomach without fluid or air by CT guidance is easy and safe, because we achieved a 100% technique rate.

De Bucourt and Tamura have been reported the method of CT fluoroscopy-guided percutaneous gastrostomy [5, 6]. But the CT fluoroscopy-guided started after insufflation by a nasal tube. In our research, all of the patients encountered the failure of the nasal tube placement due to the esophagus complete obstruction, complicate esophageal mediastinal fistula, esophageal trachea fistula or severe heart disease. The key to the success of this technology is how to puncture a stomach without fluid or air by 21 G needle and inflate the air into gastric lumen. In this study, four patients underwent the failure of stomach lumen insufflations through the 21-gauge Chiba needle puncture in the first time. The injected air exited in the abdomen cavity during the CT scan in the two cases. We guess the Chiba needle slipped from the stomach during the interval of CT scan. So, the key point of the puncture and inflation as follows: 1. Keep the patient breathing shallowly and puncture the stomach quickly. 2. CT scan is needed to confirm the position of the 21 G needle tip within the gastric cavity before insufflation, and the needle should be kept stable during the insufflation process. Once the puncture and inflation finished successfully, the remaining procedure step scan can be performed as before reported [5, 6].

In our research, the procedure was performed by CT guided, but not a real time image monitored. So, we cannot dynamically observe the diversification of air in gastric lumen during the gastropexy procedure and the trocar needle insertion. The burp or gastrointestinal peristalsis maybe decreases the gastric air and this situation could be lead the puncture difficulty and massive hemorrhage of stomach. On the contrary, under fluoroscopic guidance, the doctor can observe the stomach real time. If the air of gastric lumen flowed away, the doctor can see it and injected the air via the nasogastric tube to avoid this condition. In our experience, we injected the air via the 21 G needle intermittently during the whole procedure until the patient complained abdominal fullness. Of course, we can perform the gastrostomy under the CT fluoroscopy guidance. But this will significantly increase the dose radiation of the doctor and patient in the same situation.

In our previous experience, percutaneous fluoroscopy-guided gastrostomy has been associated with the occurrence of gastric bleeding, which at times exhibits a certain level of unpredictability and is linked to blood vessels on the stomach wall. While there were no instances of gastric bleeding complications among the 32 patients in this study, we acknowledge the possibility of gastric bleeding during the entire CT-guided gastrostomy procedure. In the event of minimal blood loss, hemostatic medication can be administered. However, substantial blood loss may necessitate surgical evaluation, based on our past treatment experiences.

Our study has some limitations and the first is due to its nature of retrospective study, there might be the potential risk for selection bias. Second, this study may have inherent bias associated with a small sample size even in two medical centers. Thus, large prospective and multi-center randomized fashion studies should be performed in the future to establish the efficacy of this method. Lastly, we did not measure the dosage of X-ray during the procedure. We cannot know which would give the less radiation injury to patient compare with the fluoroscopy guided gastrostomy. But this method can reduce the radiation dose of the doctors and nurses. Considering that it is faster to inject gas through the nasogastric tube, PFG maybe the first choice. CT-guided percutaneous gastrostomy for patients who are unsuitable or unwilling to undergo PFG.

In conclusion, we demonstrated that direct gastric puncture under whole process CT guidance is a viable, safe, and effective alternative to the patients who were considered too dangerous or impossible due to the esophagus complete obstruction, complicate esophageal mediastinal fistula, esophageal trachea fistula or severe heart disease. Of course, in some hospital there was no digital subtraction angiography (DSA), doctor also can use this method to finish the gastrostomy by CT guidance.

## Data Availability

The datasets used and/or analyzed during the current study will be available from the corresponding author on reasonable request.
