# Correction: The important role of whole-process computed tomography guidance for percutaneous gastrostomy in esophageal cancer patients who are unsuitable for or have had unsuccessful attempts with endoscopic and fuoroscopic gastrostomy

**DOI:** 10.1186/s12876-024-03165-3

**Published:** 2024-02-26

**Authors:** Xiang Geng, Qing Zhao, Hang Yuan, Hai-Liang Li, Chen-Yang Guo, Ting Yang, Wei-Jun Fan, Jung-Hoon Park, Xiao-Hui Zhao, Wen-Bo Zhu, Hong-Tao Hu

**Affiliations:** 1 Department of Minimally & Invasive Intervention, The Affiliated Cancer Hospital of Zhengzhou University, Henan Cancer Hospital, NO.127, Dongming Road, 450008 Zhengzhou, Henan Province China; 2 Department of Radiology, The Second People’s Hospital of Jiaozuo, No.17, Minzhu South Road, 454150 Jiaozuo, Henan Province China; 3 Department of Minimally & Invasive Intervention, Sun Yat-sen University Cancer Center, No.651, Dongfeng east Road, 510000 Guangzhou, Guangdong Province China; 4 Biomedical Engineering Research Center, Asan Institute for Life Sciences, Asan Medical Center, 88 Olympic-ro 43- gil, 05505 Seoul, Korea

**Correction: BMC Gastroenterol (2024) 24:14**

10.1186/s12876-023-03040-7

Following publication of the original article it was reported that there was an error in affiliation 1, namely that ‘Henan Cancer Hospital’ preceded ‘The Affiliated Cancer Hospital of Zhenzhou Univeristy’.

The original article has been updated.

